# Molecular Basis of Resveratrol-Induced Resensitization of Acquired Drug-Resistant Cancer Cells

**DOI:** 10.3390/nu14030699

**Published:** 2022-02-07

**Authors:** Chul Yung Choi, Sung-Chul Lim, Tae-Bum Lee, Song Iy Han

**Affiliations:** 1 Department of Biomedical Science, College of Natural Science, Chosun University, Gwangju 61452, Korea; blockstar@chosun.ac.kr; 2 Department of Pathology, College of Medicine, Chosun University, Gwangju 61452, Korea; sclim@chosun.ac.kr; 3 Division of Premedical Science, College of Medicine, Chosun University, Gwangju 61452, Korea; tblee01@gmail.com

**Keywords:** resveratrol, cancer, chemotherapy, drug-resistance

## Abstract

Multidrug resistance (MDR) to anticancer drugs remains a serious obstacle to the success of cancer chemotherapy. Resveratrol, a polyphenol, present in natural products exerts anticancer activity and acts as a potential MDR inhibitor in various drug-resistant cancer cells. In the process of resensitization of drug-resistant cancer cells, resveratrol has been shown to interfere with ABC transporters and drug-metabolizing enzymes, increase DNA damage, inhibit cell cycle progression, and induce apoptosis and autophagy, as well as prevent the induction of epithelial to mesenchymal transition (EMT) and cancer stem cells (CSCs). This review summarizes the mechanisms by which resveratrol counteracts MDR in acquired drug-resistant cancer cell lines and provides a critical basis for understanding the regulation of MDR as well as the development of MDR-inhibiting drugs.

## 1. Introduction

Many patients often experience recurrence of cancer after chemotherapy due to the development of multidrug resistance (MDR), which is one of the most crucial hurdles in cancer treatment [1]. MDR refers to a phenomenon wherein tumors exhibit cross-resistance to an array of drugs with different structures or action mechanisms once they become resistant to one anticancer drug [2].

Acquired drug resistance may result due to the modification of various cellular and molecular mechanisms, including (1) removal of drug by increased efflux transporters; (2) inactivation of drug due to metabolization; (3) promotion of DNA damage repair; (4) modification of drug targets; (5) regulation of cell cycle progression; (6) inhibition of programmed cell death pathways; and (7) induction of epithelial to mesenchymal transition (EMT) and cancer stem cells (CSCs) [3,4,5,6]. All these events are associated with drug resistance, either independently or in combination, and may lead to MDR through various signal transduction pathways. Therefore, understanding the mechanisms involved in drug resistance and exploring new approaches to resensitize resistant cancer cells are important for the development of improved therapeutic strategies [7].

Recently, various plant-derived compounds with anticancer properties have emerged as attractive drug candidates due to their advantages such as low toxicity and immediate availability. Among them, polyphenols, which are natural compounds present in fruits and vegetables, have been proven to have multiple benefits in the treatment of cancer as well as in several chronic diseases.

Resveratrol (3,5,4′-trans-trihydroxystilbene, RES) is abundantly produced in a wide variety of plants, such as grapes, berries, and peanuts [8], and is a major polyphenol possessing anti-inflammatory, cardiovascular protective, as well as cancer chemopreventive activities [9,10]. In particular, the prominence of RES is increasing owing to its chemosensitizing and radiosensitizing effects [11,12,13]. RES has been shown to promote the efficacy of various anticancer drugs at a low dosage [14,15] and exerts nearly no cytotoxicity in animal models [10,16,17]. More importantly, RES has been shown to sensitize various resistant cancer cells to anticancer drugs by overcoming chemoresistance mechanisms [11,18].

## 2. In Vitro and In Vivo Activity of RES in Different Tumor Models

Chemotherapeutic agents, such as vinca alkaloids (vincristine (VCR) and vinblastine), anthracyclines (doxorubicin (DOX), daunorubicin, and epirubicin), anthracenediones (mitoxantrone (MX)), antimetabolites (5-fluorouracil (5-FU), methotrexate, and gemcitabine), taxanes (paclitaxel (PTX) and docetaxel (DTX)), and platinum salts (cisplatin (CIS), carboplatin, and oxaliplatin (OXT)), are most frequently associated with drug resistance [19,20].

Due to multiple resistance responses to classical MDR drugs, the paradigm is gradually shifting toward the development of targeted anticancer drugs, which block cancer-specific pathways. The selected targeted anticancer drugs are tyrosine kinase inhibitors, including imatinib (IM), which interferes with BCR-ABL; vemurafenib (VEM), which inhibits BRAF; and cetuximab (CET) and gefitinib (GEF), which block EGFR. However, the emergence of drug resistance still seems to be an unavoidable issue [21,22]. Moreover, tamoxifen (TAM), a selective estrogen receptor (ER) modulator that has been used for all stages of ER-positive breast cancers, is also associated with acquired resistance in breast cancer cells, after long-term treatment that initially responded to antiestrogen therapy [23].

Cancer cells may eventually develop resistance to almost all types of drugs, and strategies to deal with the resistance response need to be considered during chemotherapy. However, RES has been shown to suppress acquired drug resistance caused by multiple types of anticancer drugs, which occurs in a variety of cancer tissues, including bladder, breast, colon, stomach, white blood cells, lungs, and prostate cancer [7,24,25,26]. Therefore, we tried to understand the various mechanisms involved in RES-induced reduction of acquired drug resistance. 

**Figure 1:**
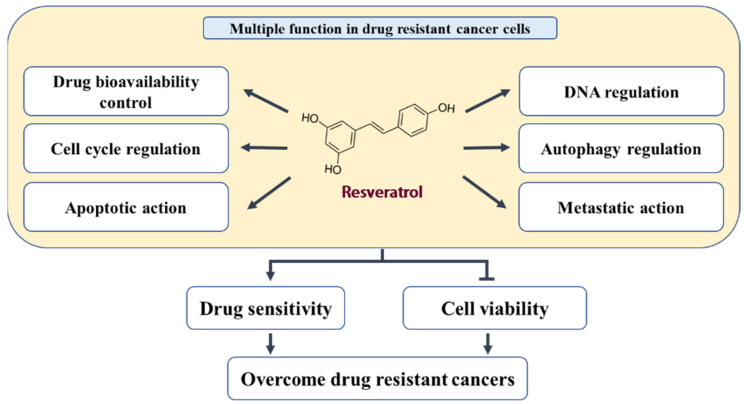
A schematic model of RES function in drug-resistant cancer cells.

For this purpose, we screened for research articles that studied the role of RES in cancer cells that acquired resistance to chemotherapeutic drugs. From 1990 to 2021, 30 reports were published (according to PubMed search results accessed on 15 December 2021, https://pubmed.ncbi.nlm.nih.gov/), that investigated the effect of RES in acquired drug-resistant cancer cells including bladder (DOX), breast (CIS, DOX, MX, PTX, TAM), colon (5-FU, CIS, OXT), gastric (DOX), leukemia (DOX, IM, VCR), lung (CIS, GEF), melanoma (VEM), oral (CET, CIS), ovarian (CIS, OXT), and prostate (DTX) cancer cells. In this review, we summarized the findings involved in resistance prevention mechanisms and the potential therapeutic role of RES in multiple drug-resistant cancer cells (Figure 1, Table 1 and Table 2).

## 3. Biological Effects and Mechanisms of RES in Acquired Drug-Resistant Cancer Cells

### 3.1. Inhibition of Drug Transporters and Drug-Metabolizing Enzymes

The overexpression of various ABC efflux transporters such as P-glycoprotein (P-gp/MDR1/ABCBl), multidrug-resistance-associated protein 1 (*MRP1*/ABCC1), breast cancer resistance protein (BCRP/ABCG2), and lung resistance protein (*LRP*) in cancer cells can significantly eliminate anticancer drugs from the cell, thus causing persistent resistance in cancer chemotherapy [27]. One way to overcome MDR is to prevent the expression or activity of ABC transporters, allowing chemotherapeutic drugs to remain in cancer cells [28]. Additionally, drug resistance can be suppressed by inhibiting the expression and activity of drug-metabolizing enzymes, including cytochrome P450s (CYPs) and glutathione-S-transferases (GSTs), resulting in altered metabolic control in cancerous cells [29].

#### 3.1.1. P-Glycoprotein (P-gp/MDR1)

In platinum-resistant cancer cells, elevated expression of MDR1 and reduced intracellular accumulation of MDR substrates have been reported. However, cellular accumulation of MDR substrates was shown to be significantly restored in the presence of RES, as compared with drug treatment alone in CIS- or OXT-resistant cancer cells. For instance, combined treatment with RES significantly elevated the cellular uptake of CIS in CIS-resistant HCT-116 colorectal cancer cells [30]. In addition, RES downregulated MDR1 expression and decreased MDR1 promoter activity in HCT116/L-OHP cells, which were established from HCT116 colorectal cancer cells to obtain resistance to OXT. Moreover, RES enhanced intracellular accumulation of the P-gp substrate rhodamine 123 (Rh123) in HCT116/L-OHP cells. The mechanism by which RES suppressed MDR1 expression in HCT116/L-OHP cells was associated with the prevention of NF-κB signaling and cAMP-responsive element binding protein (CREB) activation in an AMP-activated protein kinase (AMPK)-dependent manner [31]. Furthermore, RES significantly increased the intracellular fluorescence of Rh123 and calcein in a dose-dependent manner in P-gp-overexpressing, DOX-resistant CEM leukemia subline, CEM/ADR5000 cells. In these cells, RES exhibited a stronger inhibitory effect on P-gp than verapamil, an inhibitor of the efflux pump P-gp [32]. The combined treatment condition in which RES was added first followed by the addition of CIS 2 h later, showed significantly greater accumulation of cellular platinum and platinum–DNA adducts than the equivalent concentration of CIS treatment alone in both parental human ovarian A2780 cells and a CIS-resistant A2780 subline, A2780 cisR. The enhanced platinum drug–DNA binding and drug efficacy were considered to be mainly due to the prevention of the functioning of P-gp by RES [33].

#### 3.1.2. Multidrug Resistance-Associated Protein 1 (*MRP1*)

RES effectively reversed drug resistance in DOX-resistant pumc-91/ADM cells, established from pumc-91 bladder cancer cells. The underlying molecular basis is associated with a decrease in the expression of the ABC transporter genes, *MRP1* and *LRP*, as well as reduced levels of the drug-metabolizing enzyme, GST [24]. Similarly, combinatorial treatment with RES and DOX significantly increased the cellular accumulation of DOX compared to DOX treatment alone by downregulating the expression levels of MDR1 and *MRP1* in DOX-resistant MCF-7/ADR and HL-60/ADR cells, which were developed from MCF-7 breast cancer cells and HL-60 promyelocytic leukemia cells, respectively [34,35]. In HL-60/ADR cells, the PI3K/Akt/Nrf2 signaling pathway was suggested to play a role in DOX-mediated *MRP1* expression and drug resistance. This was supported by the observation that RES decreased the protein expression levels of PI3K, p-Akt, and Nrf-2. Furthermore, *MRP1* and PI3K inhibitors reduced the levels of p-Akt, Nrf2, and *MRP1* [35]. Moreover, RES treatment significantly increased the uptake of 5(6)-carboxyfluorescein diacetate, an *MRP1* substrate, in the DOX-resistant variant of AML-2 leukemia, AML-2/DX300 cells. Thus, RES seems to overcome the resistance of AML-2/DX300 cells through the inhibition of *MRP1* [36].

#### 3.1.3. BCRP (ABCG2)

PC9/G cells, a subline of PC9 lung cancer cells that were established to acquire resistance to GEF, have been shown to enhance the protein expression of CYP1A1 and ABCG2, thereby promoting drug metabolism and secretion. However, combined treatment with RES and GEF decreased the expression of these proteins and increased intracellular GEF accumulation compared with GEF treatment alone in PC9/G cells. Knockdown of CYP1A1 or ABCG2 increased intracellular GEF concentration, as well as enhanced the GEF-induced inhibition of EGFR phosphorylation. These results indicate the significance of drug metabolism and efflux in GEF-resistant cells, and suppression of these effects is closely associated with the antiresistance activity of RES [37]. In addition, RES has been shown to modulate BCRP-associated ATPase activity. RES significantly increased the accumulation of the established BCRP substrates, mitoxantrone (MX) and BODIPY FL prazosin, in BCRP-overexpressing, MX-resistant MCF/MR cells, derived from MCF-7 breast cancer cells but not in wild-type cells [38]. Moreover, RES stimulated vanadate-inhibitable ATPase activity in membranes, expressing BCRP, prepared from bacteria (*Lactococcus lactis*), implying possible interactions between RES and BCRP [38]. As mentioned above, RES can interfere with the expression or activity of various ABC transporters and drug-metabolizing enzymes, which may be one of the essential mechanisms in overcoming drug resistance in cancer cells.

### 3.2. Promotion of DNA Damage and Inhibition of DNA Repair and Replication 

DNA is a critical target for numerous chemotherapeutic drugs [39]. However, elevated DNA repair and tolerance to DNA damage may induce resistance to DNA-targeting drugs [40]. Inhibition of the DNA repair system may be a beneficial strategy to restore drug sensitivity in resistant cells. Notably, RES has been shown to enhance the DNA-damaging effect of anticancer drugs in several drug-resistant cancer cells. 

DNA topoisomerases are critical enzymes that maintain DNA structure during DNA replication and transcription [41]. The expression levels of DNA topoisomerase-II (Topo-II) in cancers is a predictive factor of responsiveness to therapy. However, a typical feature of MDR, induced by Topo-II-interacting drugs, is reduced Topo-II amount or activity [42,43]. The anthracycline class of drugs, such as DOX, interferes with DNA replication and induces DNA strand breaks by forming drug–Topo-II–DNA complexes in cancer cells. Although Topo-II levels were reduced in pumc-91/ADM cells, a significant increase in Topo-II was detected in the RES-treated group compared to that in the RES-untreated group [24]. Hence, RES appears to promote drug-induced DNA damage in DOX-resistant cancer cells through upregulation of Topo-II expression. In addition, RES has been shown to enhance the DNA damaging effect in 5-FU-R cells, established from 5-FU-sensitive HCT116 cells to acquire resistance to 5-FU. 5-FU-R cells highly expressed the 5-FU-resistance protein, thymidylate synthase, and anti-apoptotic proteins, such as FLICE-like inhibitory protein, DNA polymerase eta (POL-H), DNA polymerase beta (POL-β), DNA damage-binding protein 2 (DDB2), and flap endonuclease 1 (FEN1), in comparison to parental HCT116 cells. As expected, upon exposure to a synthetic DNA damaging agent, 1,3-bis(2-chloroethyl)-1-nitrosourea (BCNU), 5-FU-R cells were highly resistant. However, a combination of RES and BCNU significantly increased the sensitivity and DNA damage of 5-FU-R cells; inhibited DNA repair proteins, POL-H, POL-β, FEN1, and DDB2; and increased the expression of the DNA damage response protein, adenomatous polyposis coli (APC). DNA damage and apoptosis were elevated with increasing concentrations of RES and a constant BCNU concentration, suggesting the critical role of RES in the sensitization of 5-FU-R cells [44]. 

The inhibitory effects of RES in the repair of DNA double-strand breaks have also been reported in CIS-resistant cancer cells. Platinum drugs, such as CIS, induce DNA damage by forming platinum–DNA adducts that interfere with DNA replication and transcription. CIS treatment increased the levels of Rad51 protein, which is essential for the homologous recombination repair of DNA double-strand breaks in human breast cancer MCF-7 cells and the CIS-resistant subline, MCF-7R cells. However, RES decreased the relative levels of Rad51 and the transcript levels of homologous recombination initiation complex components (*Nbs-1*, *Mre-11*, and *Rad-50*) and increased H2AX (p-serine139) levels, which are used as a marker for DNA damage. These results indicate that RES, in combination with CIS, suppresses the expression or activity of DNA repair proteins, thereby inhibiting DNA damage repair [7].

### 3.3. Cell Cycle Regulation

Cell cycle arrest may be ambivalent in determining the cancer cell’s fate in response to chemical drugs. Inhibition of the cell cycle may result in a relative insensitivity to drugs and act as a defense mechanism since cells become less responsive to toxic stimuli in their resting phases [45]. Conversely, deregulation of the cell cycle also acts to enhance the sensitivity of resistant cells to chemotherapy since blockage of cell cycle progression often escapes alternative cell death. In the most studies, cell cycle arrest induced by RES in drug-resistant cancer cells was observed along with the activation of apoptosis, which is proved by an increase in apoptotic signal molecules, including PTEN, p53, and active caspases-3, -7, -8, and -9, and a decrease in anti-apoptotic regulators, such as p-AKT and EGFR [16,37,44,46,47]. 

Therefore, RES-induced cell cycle arrest may proceed toward apoptosis induction and overcoming drug resistance rather than acting as a defense mechanism. Progression of the cell cycle is regulated by cyclin-dependent kinases (CDKs), cyclins, and Cdk inhibitors (CDKIs). CDKs are upregulated by cyclins (A, B, D, and E) and downregulated by CDKIs [48]. 

RES has been shown to inhibit cell cycle progression in drug-resistant cancer cells as well as in parental cancer cells, including colon, breast, and prostate cancer cells [10,16,49,50]. Indeed, RES arrested the cell cycle in the G0–G1 phases in SPC-A-1/CDDP cells, which are generated from SPC-A-1 lung cancer cells to acquire resistance to CIS [16]. In addition, RES significantly induced G1 arrest in TAM-resistant breast cancer cells, MCF-7 TR1, as well as in parental MCF-7 cells by increasing p53-dependent p21 expression. In a study using MCF-7 TR1 cells, the sustained activation of p38MAPK by RES was suggested to be a critical mechanism in the modulation of p53 and ERα expression. Moreover, the expression of cyclin D1 and the estrogen-regulated gene, *IRS1*, was significantly decreased by RES treatment [51]. 

Furthermore, RES has been shown to act as a potential chemosensitizer in MCF-7-ADR cells, which are DOX-resistant MCF-7 breast cancer cells. During this process, RES increased the expression of miR-122-5p, and both RES and miR-122-5p mimic significantly downregulated CDKs (CDK2, CDK4, and CDK6) and induced G1 arrest in MCF-7-ADR cells. However, inhibitors of miR-122-5p significantly reversed the effects of RES. Thus, it has been suggested that miR-122-5p is involved in RES-mediated cell cycle arrest in a CDK-dependent manner [46].

Certain drugs, such as CIS, have also been reported to induce cell cycle arrest through p21 induction in multiple cancer cells. In parental MCF-7 and CIS-resistant MCF-7_R_ cells, treatment with CIS with or without RES as well as treatment with RES alone upregulated *p21* gene expression levels. Notably, combined treatment with CIS and RES resulted in further increase in p21 expression and sensitized CIS-induced resistance in MCF-7_R_ cells [47]. The synergistic effects of RES and CIS were consistently observed in HCT116 colorectal cancer cells. Simultaneous treatment with CIS and RES resulted in a significant increase in the percentage of cells in the G_0_ phase in parental and CIS-resistant HCT-116 cells [30]. RES was also reported to affect cell cycle regulation in 5-FU-R, which are 5-FU-resistant HCT-116 cells. In this cell line, combined treatment with BCNU and RES significantly intensified the increase in cell cycle inhibitory protein p21 and phosphatase and tensin homolog (PTEN) and the decrease in cyclin-dependent kinase-1 (CDC2) as the concentration of RES increased [44].

In contrast to several reports showing G0 or G1 arrest by RES, some studies have revealed that RES interferes with S-G2/M transition in certain drug-resistant cancer cells, leading to the accumulation of cells in the S or G2/M phase. In pumc-91/ADM cells, cell proliferation gradually decreased as the concentration of RES increased due to the blockage of cell cycle progression. Analysis of the total cell population indicated that RES treatment led to cell cycle arrest at the S phase, accompanied by a decrease in the number of cells in the G1 phase in pumc-91/ADM cells [24]. In HL60/VCR cells, a subline of promyelocytic leukemia HL60 cells that are resistant to VCR, RES inhibited cell cycle progression at the S phase. VCR exerts antitumor activity by inhibiting microtubule assembly and primarily interferes with the mitotic spindle, thereby arresting the cell cycle in the metaphase of mitosis. The mechanism of VCR resistance is generally associated with an increase in drug transporters since VCR acts as a substrate of MDR transporters. Hence, cell cycle arrest by co-administration of RES can be considered to be associated to the amplified action of VCR through drug-efflux inhibition. However, combined treatment with RES intensified cell cycle arrest in the S phase and sensitized HL60/VCR cells, which are P-gp positive, as well as P-gp-negative parental HL60 cells to several drugs, such as DOX, cycloheximide, busulfan, gemcitabine, and PTX [52]. Thus, there is possibility that RES contributes to the sensitization of HL60 cells by inducing cell cycle arrest through pathways both dependent on and independent of P-gp inhibition. RES also affected cell cycle regulation in PC9/G cells, which are GEF-resistant non-small-cell lung cancer (NSCLC) cells. RES alone significantly increased the percentage of cells in the G2/M phase, and co-treatment with RES and GEF further elevated G2/M phase arrest compared to either treatment alone. This was accompanied by increased expression of p53 and p21 in PC9/G cells [37]. Considering these results together, cell cycle arrest by RES may be associated with alternative cell death since it deprives the cells of their proliferative capacity, thereby overcoming drug resistance.

### 3.4. Pro-Apoptotic and Antisurvival Actions 

Induction of cell death and suppression of cell survival are fundamental principles of chemotherapy [53]. Chemotherapeutic drugs can initiate apoptosis, the major type of programmed cell death in cancer cells. The extrinsic and intrinsic pathways are well-known apoptotic processes, which ultimately activate the cysteine proteases (caspases), which are critical apoptotic executioners [54]. During the apoptotic process, the efficiency generally depends on prosurvival signaling factors, such as Akt and ERK1/2, and apoptosis regulatory proteins, such as Bcl-2 family members [55]. RES has been reported to affect multiple pro-apoptotic and antisurvival regulators in drug-resistant cancer cells.

#### 3.4.1. p53 Induction

RES has been shown to upregulate the essential pro-apoptotic factor, p53 in MCF-7 breast cancer cells resistant to CIS (MCF-7_R_) and TAM (MCF-7/TR1) and PC9 lung cancer cells resistant to GEF (PC9/G) [37,47,56]. Resistance in both MCF-7 and MCF-7_R_ cells has been associated with the downregulation of p53 [47]. Induction of p53 by RES may enhance apoptosis, either by inducing the expression of pro-apoptotic genes or by regulating the transcription-independent mitochondrial pathway, which is the commonly known function of p53. Studies have indicated that RES activates kinases such as casein kinase 1 (CK1), checkpoint kinase 2 (CHK2), and AMPK to induce phosphorylation of p53 at serine20, which is required to activate p53 and, thereby, induce *Bax* and *PUMA* genes [47]. 

#### 3.4.2. Caspase Activation

In 5-FU-resistant HCT116 cells, RES alone can induce caspase-3 cleavage, and it further enhances caspase-3 activity in combination with BCNU [44,57]. In addition, combinatorial treatment with RES has been reported to increase caspase-3 cleavage in several resistant cancer cells, including PC9/G cells [37]; MCF-7/TR cells [56]; MCF-7/DOX, a DOX-resistant subline of MCF-7 cells [58]; and SGC7901/DOX, a DOX-resistant subline of SGC7901 gastric cancer cells [26]. Moreover, RES-enhanced activation of caspase-3 and caspase-9 was observed in SPC-A-1/CDDP cells [16], and RES-enhanced activation of caspase-8 and caspase-9 was observed in MCF-7-ADR, another DOX-resistant subline of MCF-7 cells [46]. RES was also able to induce apoptosis in MDA-MB-231/PacR cells, which are established from MDA-MB-231 breast cancer cells to acquire PTX resistance, via a mechanism that was associated with decreased survivin expression and increased caspase-7 activation [59].

#### 3.4.3. Inhibition of ERK and AKT Pathways

RES appears to exert its anticancer activity by suppressing cell survival signaling pathways, such as ERK and AKT. The ERK pathway is the major target pathway for drugs such as CET, used in the treatment of EGFR-mutated cancer. In CET-resistant cancer cell lines, SAS-R, Sa3-R, and HSC-3-R, generated from SAS, Sa3, and HSC-3 oral cancer cells, respectively, part of the mechanism responsible for CET resistance has been identified as the urokinase-type plasminogen activator receptor (uPAR)/integrin β1/Src/FAK signal circuit that converges with ERK1/2 phosphorylation [60]. This signaling pathway enables the induction of CET resistance by activating ERK in the absence of overexpression or activating mutations of EGFR. The overexpression of uPAR has been found to be a major factor in inducing CET resistance, and combination with RES inhibits the expression of uPAR and integrin β1. Moreover, RES inhibited ERK1/2, downstream of EGFR, which exhibits additive effects in preventing CET resistance in vitro and in vivo [60]. RES-induced apoptosis was also mediated by the suppression of Akt and ERK1/2 activities. In TAM-resistant MCF-7/TR cells, phosphorylation of Akt and ERK1/2 was reduced in an RES-dose-dependent manner [56]. Similarly, RES suppressed the PI3K/Akt/mTOR signaling pathway in MCF-7/DOX cells [58] and p-Akt levels in VEM-resistant melanoma cells [61].

#### 3.4.4. Inhibition of NF-κB

Activation of the pro-inflammatory NF-κB signaling pathway is a critical event in tumor progression and chemoresistance [62]. RES-induced apoptosis is in part related to the downregulation of NF-κB activation through the inhibition and degradation of IκBα kinase in parental HCT116 cells and 5-FU-resistant HCT116R cells [57]. Similarly, RES suppresses TNF-β-enhanced survival of HCT-116R cells by promoting apoptosis via blocking NF-κB (p65) activation and NF-κB-dependent gene products [25].

#### 3.4.5. Alteration of Bcl-2 Family Members

RES has also been shown to modify the levels of mitochondrial Bcl-2 family proteins. Alteration of the Bcl-2/Bax ratio has been detected in CIS-resistant MCF-7_R_ cells. Upon RES treatment, the levels of pro-apoptotic protein Bax increased while the levels of antiapoptotic protein Bcl-2 decreased, thereby restoring apoptosis [47]. In CIS-resistant CAR cells, which are developed from CAR human oral cancer cells, RES has been observed to increase the protein levels of Bax and Bad and decrease the protein levels of Bcl-2 and the phosphorylation of Bad on serine 136 [63]. The phosphorylation of Bad is regulated by the PI3K/Akt pathway and promotes cell survival by interacting with 14-3-3t protein, which sequesters Bad from Bcl-xL [64]. Moreover, RES increased the Bax/Bcl-xL ratio in 5-FU-R cells [44] and decreased Bcl-2 levels in pumc-91/ADM cells in the process of promoting apoptosis [24]. Furthermore, RES reduced the expression of Bcl-2 in MCF-7-ADR cells through the overexpression of miR-122-5p [46].

#### 3.4.6. Prevention of Clusterin Expression

PC3-DR and DU145-DR cells are resistant to DTX and are developed from PC3 and DU145 prostate cancer cell lines, respectively. These cells have been found to be resistant to tumor necrosis factor–related apoptosis-inducing ligand (TRAIL), although they express TRAIL receptors, such as TRAIL receptor-1 and TRAIL receptor-2. Upregulation of clusterin expression has been shown to be associated with TRAIL resistance in PC3-DR and DU145-DR cells. Notably, RES prevented clusterin expression by suppressing Src/Jak activation and subsequent Stat1 phosphorylation, thereby sensitizing DTX-resistant tumor cells to TRAIL [65].

#### 3.4.7. Other Factors

In addition to those mentioned above, RES has been suggested to affect various other factors involved in apoptotic regulation. RES can enhance apoptosis by increasing the levels of PTEN, tuberous sclerosis complex 1 (TSC1), and TSC2 in SGC7901/DOX cells [26], augmenting the protein levels of Apaf-1, AIF, and Endo G in CIS-resistant CAR cells [63] and suppressing the expression of survivin in MDA-MB-231/PacR and SPC-A-1/CDDP cells [16,59].

### 3.5. Autophagy Regulation

Autophagy is an evolutionarily conserved adaptive mechanism that enables cells to maintain homeostasis and survive stressful environments by facilitating the degradation and recycling of cytoplasmic constituents and organelles [66]. The autophagic process is initiated by phagophore assembly, autophagosome formation, and fusion of the autophagosomes with lysosomes, leading to lysosomal degradation of autophagosomal contents by lysosomal acid hydrolases [67,68]. These processes are tightly regulated by distinct signaling pathways that control autophagy-related (ATG) proteins [69]. The role of autophagy in the treatment of cancer MDR can be both beneficial and harmful. It contributes to the development of MDR and protects cancer cells from cytotoxic drugs but also kills drug-resistant cells in which apoptotic pathways are disabled [3]. In the latter case, autophagy acts as a death executioner to trigger a form of type II programmed cell death, which utilizes a signal pathway distinct from type I programmed cell death, apoptosis [53].

RES has been reported to induce autophagic cell death through the modulation of AMPK and Akt signaling in CIS-resistant CAR cells. Exposure to RES increased the protein levels of AMPKα and phosphorylated AMPKα at Thr172 but decreased the phosphorylation of Akt on Ser473 and of mTOR on Ser2448. It has also been shown to increase the protein levels of key autophagy markers such as Atg5, Atg7, Atg12, Atg14, Atg16L1, Beclin-1, and microtubule-associated protein 1 light chain 3 (LC3)-II and to decrease the protein levels of Rubicon, a negative regulator of autophagy [63].

RES-induced LC3-II accumulation has also been observed in several other cancer cells, including IM-sensitive and IM-resistant chronic myelogenous leukemia (CML) cells [70]; human ovarian A2780 and CIS-resistant subline, A2780CP cells [71]; and PC9/G cells [37]. RES treatment resulted in the loss of cell viability and antileukemic effects via the induction of apoptosis and autophagic death in IM-sensitive and IM-resistant CML cells. However, in these cells, loss of cell viability is only partly affected in the presence of the pan-caspase inhibitor, z-VAD-fmk, and mainly due to autophagic death. RES-triggered autophagy is controlled by increased expression of ATG3, JNK-dependent accumulation of p62, and inhibition of the mTOR pathway [70].

Therefore, RES may regulate autophagy-related molecules (ATGs, beclin, LC3, and Rubicon) and trigger autophagic cell death by interfering with the PI3K/Akt/mTOR pathway, AMPK activation, and JNK-mediated p62 expression in drug-resistant cancer cells.

### 3.6. Inhibition of EMT and CSCs

EMT plays an essential role not only in cancer cell invasion and metastasis but also in drug resistance. Numerous EMT-related signaling pathways are associated with drug resistance in cancer cells [72]. The critical hallmarks of EMT are loss of E-cadherin expression and upregulation of vimentin, snail, and slug proteins [73]. CSCs, a subset of cancer cells possessing self-renewal capacity, are also responsible for the development of resistance to anticancer drugs [74]. CD44, CD133, epithelial-specific antigen, and aldehyde dehydrogenase 1 (ALDH1) are markers of CSCs [75]. EMT and CSCs remain under control at the gene level by multiple signaling pathways, such as the MEK/ERK, TGF-β/SMAD, JAK/STAT, PI3K/Akt/NF-κB, and the WNT/β-catenin pathway [76]. Moreover, some populations of CSCs share properties with EMT-like cells [77]. Targeting EMT and CSCs has been recognized as a potential therapeutic strategy to overcome chemoresistance, and RES has been shown to reverse EMT and CSC features in several drug-resistant cancer cells. 

In MCF7/DOX cells possessing DOX resistance, combinatorial treatment with RES and DOX inhibited DOX-induced cell migration, invasion, and metastasis [58]. RES reversed EMT properties by upregulating SIRT1 and downregulating vimentin, N-cadherin, and β-catenin in MCF7/ADR cells [78]. Similarly, in SGC7901/DOX cells, RES antagonized DOX-induced EMT by downregulating vimentin and β-catenin and upregulating E-cadherin, thereby preventing cell migration [26]. RES also affects TGF-β-related signaling during the acquisition of EMT- and CSC-like features. For instance, TAM-resistant MCF-7/TR cells undergo EMT driven by enhanced endogenous TGF-β/Smad signaling. However, RES restored the expression of epithelial markers such as E-cadherin and γ-catenin and downregulated the expression of mesenchymal markers, including fibronectin, vimentin, and N-cadherin. Furthermore, RES suppressed TGF-β production and the phosphorylation of Smad2 and Smad3 in these cells [56].

On the other hand, the invasive ability of HCT116 colorectal cancer cells cultured in 3D alginate matrix was increased in the presence of TNF-α or TNF-β. However, addition of RES dramatically suppressed TNF-α- or TNF-β-induced survival and invasion of parental HCT116 and 5-FU-resistant HCT116 (HCT116R) cells. In addition, RES sensitized TNF-β-enhanced chemoresistance of HCT116R cells to 5-FU. This study showed that RES decreased TNF-β-induced expression of CSC markers (CD133, CD44, and ALDH1) and the activation of tumor-promoting factors (NF-κB(p65), MMP-9, and CXCR4) in both parental HCT116 and HCT116R cells. RES also reduced vimentin and slug levels and elevated E-cadherin expression in both cell lines [25]. Thus, RES may contribute to the inhibition of CSCs induced by TNF-α and TNF-β, as well as the anticancer drug, 5-FU. Another study showed that RES promoted the transition of 5-FU-induced formation of microvilli to a planar cell surface, which occurred concurrently with the upregulation of desmosomes, gap and tight junctions, and E-cadherin expression, and attenuated drug resistance by preventing EMT factors including vimentin, slug, and MMP-9 in HCT116 and 5-FU-resistant HCT116R cells [57]. Prevention of EMT-like characteristics by RES has also been observed in CIS-resistant cancer cells. RES reduced the migration and invasive capacity of CIS-resistant CAR cells by inhibiting the phosphorylation of ERK and p-38MAPK as well as suppressing MMP2 and MMP9 expression [79]. Likewise, RES reversed CIS-induced snail expression by blocking the ERK pathway, thereby inhibiting the morphological changes and cell migration in ovarian cancer A2780 and A2780CP (resistant to CIS) cells [71]. 

## 4. Conclusions

Since MDR of cancer is a complex phenomenon caused by multiple pathological mechanisms, the effective treatment option to overcome MDR might be combination therapy. Notably, RES has been found to be a potent agent to reverse drug resistance by targeting multiple genes and pathways involved in cancer cell survival and malignancy. Considering the studies summarized in this review, RES has been shown to be capable of (1) decreasing drug-metabolizing enzymes and drug transporters, (2) enhancing DNA damage and suppressing DNA repair and replication, (3) inducing cell cycle arrest, (4) promoting apoptotic and autophagic cell death, and (5) inhibiting EMT and CSC formation in drug-resistant cancer cells. In particular, it is important to note that RES itself has low toxicity, and combinatorial treatment with anticancer drugs has effectively promoted the efficacy of anticancer drugs both in vitro and in vivo. Various preclinical studies have reported the anticancer properties of RES in xenografts and benzo-(a)pyrene-induced mouse models of cancer. In multiple clinical trials, the intake of approximately 0.5–1 g of resveratrol has exhibited anticarcinogenic effects, such as modulation of carcinogen-metabolizing enzyme systems or increase of apoptosis in malignant hepatic tissues, with relative safety. Based on these studies, RES has been suggested to be a strong candidate to overcome the increasing prevalence of acquired drug resistance in cancer chemotherapy, with emphasis on enhancing their preventive and therapeutic properties. However, it still requires further clinical evaluation since there has been a case with an unacceptable safety profile and minimal efficacy when administered at high doses to patients with relapsed or refractory multiple myeloma. In conclusion, RES, in combination with anticancer drugs, represents a promising strategy for the treatment of acquired drug-resistant cancers that are no longer susceptible to current treatments.

**Table 2:** Assessed parameters and outcome measures of RES treatment in various drug-resistant cancer cells.

Cancer	Selecting Drug	Resistant Cells	Assessed Parameter	Outcome Measures	Reference
Bladder	Doxorubicin	Pumc-91/ADM	↑Topo-II, ↓Bcl-2, ↓GST, ↓LRP, ↓*MRP1*	↑S arrest, ↓Proliferation	[24]
Breast	Cisplatin	MCF-7R	↑γ-H2AX, ↓Rad51	↓Proliferation, ↓Repair of DNA damage	[7]
		MCF-7_R_	↑AMPK, ↑Bax, ↑CHK2, ↑CK1, ↑p21, ↑PUMA, ↑p-p53 (Serine20), ↓Bcl-2, ↓p-p53 (Serine15 and 46)	↑Apoptosis, ↓Proliferation	[47]
	Doxorubicin	MCF-7-ADR	↑Caspase-8, ↑Caspase-9, ↑miR-122-5p ↓Bcl-2, ↓CDK2, ↓CDK4, ↓CDK6	↑Apoptosis, ↑G1 arrest, ↓Proliferation	[46]
		MCF7/ADR	↑SIRT1, ↓β-Catenin, ↓N-Cadherin, ↓Imentin	↑Apoptosis, ↓Cell migration, ↓EMT, ↓Proliferation	[78]
		MCF-7/DOX	↑Caspase-3, ↓PI3K, ↓p-Akt/Akt, ↓p-mTOR/mTOR	↑Apoptosis, ↓Cell migration, ↓Cell invasion, ↓Metastasis, ↓Proliferation, ↓Tumor volume	[58]
		MCF-7/adr	↓MDR1, ↓*MRP1*	↑Cellular influx, ↓Proliferation, ↓Tumor volume	[34]
	Mitoxantrone	MCF/MR	-	↑ATPase activity, ↑Cellular accumulation	[38]
	Paclitaxel	MDA-MB-231/PacR	↑Caspase-7, ↓Survivin	↑Apoptosis, ↑Senescence ↑Sub-G1 arrest, ↓Colony formation ↓Proliferation	[59]
	Tamoxifen	MCF-7/TR	↑Caspase-3, ↑E-Cadherin, ↑γ-Catenin, ↓Fibronectin, ↓N-Cadherin, ↓p-Akt, ↓p-ERK, ↓p-Smad2, ↓p-Smad3, ↓TGF-β, ↓Vimentin	↑Apoptosis, ↓EMT	[56]
		MCF-7 TR1	↑p21, ↑p53, ↓Cyclin D1, ↓ERα, ↓IRS1	↑G1 arrest, ↓Proliferation	[51]
Colon	5-FU	HCT-116R	↑Caspase-3, ↑E-Cadherin, ↓ALDH1, ↓CD133, ↓CD44, ↓CXCR4, ↓MMP-9, ↓p-NF-kB, ↓Slug, ↓Vimentin	↑Apoptosis, ↓Cell invasion, ↓CSC, ↓EMT, ↓Metastasis	[25]
		HCT116R	↑Caspase-3, ↑E-Cadherin, ↓MMP-9, ↓p-IkBa, ↓p-NF-kB, ↓Slug, ↓Vimentin,	↑Apoptosis, ↑Intracellular junction, ↓Cell invasion, ↓EMT, ↓Metastasis, ↓Proliferation	[57]
		5-FU-R	↑APC, ↑Bax/Bcl-xL, ↑Caspase-3, ↑p21, ↑PTEN ↓CDC-2, ↓DDB2, ↓FEN-1, ↓POLH, ↓POL-β	↑Apoptosis, ↑DNA damage, ↑Sub-G1 arrest, ↓Proliferation	[44]
	Cisplatin	CIS-resistant HCT-116 CRC	-	↑Apoptosis, ↑Cellular uptake, ↑G0 arrest, ↓Proliferation	[30]
	Oxaliplatin	HCT116/L-OHP	↑AMPK, ↓MDR1, ↓p-CREB, ↓p-NF-kB	↑Cellular uptake, ↓Proliferation	[31]
Gastric	Doxorubicin	SGC7901/DOX	↑Caspase-3, ↑Caspase-9, ↑E-Cadherin, ↑TSC1, ↑TSC2, ↓p-mTOR, ↓p-Akt, ↓β-Catenin, ↓Vimentin	↑Apoptosis, ↓Cell migration, ↓EMT, ↓Proliferation, ↓Tumor volume	[26]
Leukemia	Doxorubicin	CEM/ADR5000	-	↑Apoptosis, ↑Cellular uptake, ↓Proliferation	[32]
		HL-60/ADR	↓*MRP1*, ↓Nrf2, ↓p-Akt, ↓PI3K	↑Cellular uptake, ↓Proliferation	[35]
		AML-2/DX300	↓*MRP1*	↑Apoptosis, ↑Cellular uptake, ↓Proliferation	[36]
	Imatinib	IM-R K562	↑Atg3, ↑LC3-Ⅱ, ↑p62/SQSTM1, ↑p-AMPK, ↑p-JNK, ↓p-mTOR	↑Autophagy, ↓Colony formation, ↓Proliferation	[70]
	Vincristine	HL60/VCR	-	↑S arrest, ↑sub-G1 arrest, ↓Proliferation	[52]
Lung	Cisplatin	SPC-A-1/CDDP	↑Caspase-3, ↓Survivin	↑Apoptosis, ↑G0-G1 arrest, ↓Proliferation, ↓Tumor volume	[16]
	Gefitinib	PC9/G	↑Caspase-3, ↑LC3B-II, ↑p21, ↑p53, ↓p-EGFR, ↓ABCG2, ↓CYP1A1	↑Apoptosis, ↑Autophagy, ↑Cellular uptake, ↑G2/M arrest, ↑Senescence	[37]
Melanoma	Vemurafenib	VEM resistant	↓p-Akt	↓Proliferation	[61]
Oral	Cetuximab	SAS-R, Sa3-R, HSC-3-R	↓Integrin β1, ↓p-ERK1/2, ↓p-FAK, ↓uPAR	↓Proliferation, ↓Tumor volume	[60]
	Cisplatin	CAR	↑AIF, ↑Apaf-1, ↑Atg5, ↑Atg7, ↑Atg12, ↑Atg14, ↑Atg16L1, ↑Bad, ↑Bax, ↑Beclin-1, ↑Caspase-3, ↑Caspase-9, ↑Endo G, ↑LC3-Ⅱ, ↑p-AMPKα, ↓p-AKT, ↓p-mTOR, ↓Bcl-2, ↓p-Bad, ↓Rubicon	↑Apoptosis, ↑Autophagy, ↓Proliferation	[63]
			↓MMP-2, ↓MMP-9, ↓p-ERK, ↓p-p38	↓Cell migration ↓Cell invasion	[79]
Ovarian	Cisplatin	A2780CP	↑LC3B-Ⅱ, ↓p-ERK, ↓Snail,	↑Autophagy, ↑Cell death, ↑Senescence, ↓Cell migration, ↓EMT	[71]
		A2780^cisR^	-	↑Cellular accumulation, ↑Platinum–DNA binding, ↓Proliferation	[33]
Prostate	Docetaxel	PC3-DR	↑Caspase-3, ↓Clusterin, ↓p-Jak, ↓p-Src, ↓p-Stat1	↑Apoptosis	[65]

↑: upregulation, ↓: downregulation.

## Figures and Tables

**Table 1:** RES-induced regulation of cellular signaling molecules and gene regulatory factors in various drug-resistant cancer cells.

Target	Regulatory Molecules		Cellular Effect	
	↑ Upregulation	↓ Downregulation	↑ Upregulation	↓ Downregulation
Drug transporters and drug-metabolizing enzymes	AMPK	ABCG2, GST, LRP1, MDR1, *MRP1*, Nrf2, p-AKT, p-CREB, p-NF-κB, PI3K	Cellular accumulation	ABC transporters ATPase activity Detoxification
DNA damage, repair, and replication	APC, Topo-II, γ-H2AX	DDB2, FEN-1, POLH, POL-β, Rad51	DNA damage	DNA repair DNA replication
Cell cycle regulation	miR-122-5p, p21, p53, PTEN	CDC2, CDK2, CDK4, CDK6, Cyclin D1, ERα, IRS1	Cell cycle arrest	-
Pro-apoptotic and anti-apoptotic action	AIF, AMPK, Apaf-1, Bad, Bax, Caspae-3, Caspase-7, Caspase-8, Caspase-9, CHK2, CK1, Endo G, miR-122-5p, p53, p-p53(S20), PTEN, PUMA, TSC1, TSC2	Bcl-2, Bcl-xL, Clusterin, Integrin β1, p-AKT, p-Bad(s136), p-EGFR, p-ERK1/2, p-FAK, PI3K, p-IkBα, p-Jak, p-mTOR, p-NF-κB, p-p53(S15, S46), p-Src, p-Stat1, Survivin, uPAR	Apoptosis Cell death Senescence Sub-G1 arrest	Cell proliferation Tumor volume
Autophagy regulation	Atg3, Atg5, Atg7, Atg14, Atg12, Atg16L1, Beclin-1, LC3-Ⅱ, p62, p-AMPKα, p-JNK	p-AKT, p-mTOR, Rubicon	Autophagy	-
Migration, invasion, metastasis, EMT, and CSC	E-cadherin, SIRT1, γ-catenin	ALDH1, CD133, CD44, CXCR4, Fibronectin, MMP-2, MMP-9, N-Cadherin, p-ERK, p-NF-κB, p-p38, p-Smad2, p-Smad3, Slug, Snail, TGF-β, Vimentin, β-Catenin	Intracellular junction	Cell migration, invasion, and metastasis Colony formation CSC EMT

## Data Availability

Not applicable.
